# Nonclinical Development of BCG Replacement Vaccine Candidates 

**DOI:** 10.3390/vaccines1020120

**Published:** 2013-04-16

**Authors:** Kamalakannan Velmurugan, Leander Grode, Rosemary Chang, Megan Fitzpatrick, Dominick Laddy, David Hokey, Steven Derrick, Sheldon Morris, David McCown, Reginald Kidd, Martin Gengenbacher, Bernd Eisele, Stefan H.E. Kaufmann, John Fulkerson, Michael J. Brennan

**Affiliations:** 1 Aeras, Rockville, MD 20850, USA; 2 Vakzine Projekt Management GmbH, Mellendorferstrasse 9, 30625 Hannover, Germany; 3 Center for Biologics Evaluation and Research, Food and Drug Administration, Bethesda, MD 20892, USA; 4 Max Planck Institute for Infection Biology, Department of Immunology, Charitéplatz, 110117 Berlin, Germany

**Keywords:** tuberculosis, live vaccines, *Mycobacterium tuberculosis*, recombinant BCG

## Abstract

The failure of current *Mycobacterium bovis* bacille Calmette–Guérin (BCG) vaccines, given to neonates to protect against adult tuberculosis and the risk of using these live vaccines in HIV-infected infants, has emphasized the need for generating new, more efficacious and safer replacement vaccines. With the availability of genetic techniques for constructing recombinant BCG (rBCG) strains containing well-defined gene deletions or insertions, new vaccine candidates are under evaluation at both the preclinical and clinical stages of development. Since most BCG vaccines in use today were evaluated in clinical trials decades ago and are produced by outdated processes, the development of new BCG vaccines offers a number of advantages that include a modern well-defined manufacturing process along with state-of-the-art evaluation of safety and efficacy in target populations. We provide a description of the preclinical development of two novel rBCGs, VPM1002 that was constructed by adding a modified *hly* gene coding for the protein listeriolysin O (LLO) from *Listeria monocytogenes* and AERAS-422, which carries a modified *pfoA* gene coding for the protein perfringolysin O (PFO) from *Clostridium perfringens*, and three genes from *Mycobacterium tuberculosis*. Novel approaches like these should be helpful in generating stable and effective rBCG vaccine candidates that can be better characterized than traditional BCG vaccines.

## 1. Introduction

Although still one of the most widely used vaccines in the world, BCG originally developed by Calmette and Guérin more than 90 years ago and first administered to infants in 1921 [1], has not been effective in reducing the global burden of tuberculosis (TB). Further, HIV-infected infants are at greater risk of developing disseminated BCG infections (BCGosis) [2] following immunization, which is a risk in countries like South Africa with high levels of both diseases. BCG vaccines still remain in use because they are associated with protection against childhood TB and reduce the incidence of extrapulmonary (meningeal and miliary) forms of TB in infants. Therefore, a safer and more effective BCG replacement vaccine will reduce the global burden of TB with minimized risk to HIV-TB co-infected individuals. Of the several new TB vaccine candidates that have been studied in human clinical trials over the past decade, three have been recombinant BCG vaccines altered by modern molecular technology to potentially be safer, more immunogenic and efficacious [3]. Two of these rBCG vaccines are described in more detail in this report. 

The rBCG vaccine, VPM1002 was originally developed by Stefan H.E. Kaufmann and his team at the Max Planck Institute for Infection Biology in Berlin [4,5]. Two genetic modifications were introduced simultaneously into the BCG Danish (Sub type Prague) genome; these included integration of a gene *hly* from *Listeria monocytogenes* coding for the protein LLO, and inactivation of BCG’s gene for urease subunit C (*ureC*) to allow the acidification of the phagosomal compartment. Vakzine Projekt Management, GmbH (VPM) has facilitated the further development and acts as a sponsor for the clinical testing of this novel rBCG vaccine. 

The non-profit product development organization, Aeras, has sponsored the development of two rBCGs that have entered clinical trials. The first rBCG vaccine, rBCG30, overexpressed a single *Mycobacterium tuberculosis* (Mtb) antigen (Ag85B) in BCG Tice. This vaccine was shown to demonstrate substantially better protection *vs*. Mtb challenge in an animal model compared to its parental BCG strain, and elicited Ag85B specific CD4 T cell responses in humans [6]. Although this vaccine was found to be safe and immunogenic in a phase I clinical trial [7], it was not pursued further due to the presence of an antibiotic resistance marker. In a separate development rBCG strain, AERAS-422 was constructed using BCG Danish-SSI 1331 as a platform, to express a mutated *pfoA* gene coding for the protein perfringolysin O (PFO) from *Clostridium perfringens*, with a mode of action similar to listeriolysin [8]. It also overexpressed three Mtb antigens (Ag85A, Ag85B and Rv3407).

In this review, we provide the details on the developmental strategies used to design and characterize these two novel rBCG vaccines and in addition, summarize the methods and tests used to manufacture and evaluate the candidate products to meet regulatory requirements and describe the pre-clinical assays developed to characterize the critical parameters of these live TB vaccine candidates. Although pharmacopeial guidelines exist for the testing and evaluation of traditional BCG vaccines [9], differences in rBCGs, manufacturing procedures and nonclinical testing of these genetically modified constructs demand new approaches for preclinical evaluation and manufacturing. This article provides a comparison between the modern methods being used to generate and test these new rBCG vaccines and the traditional methods used to generate BCG vaccines. The development of safer BCG replacement vaccines could provide a vaccine that might operate through a mechanism supported by rational design, manufactured by modern cGMP methods and evaluated according to modern cGCP clinical trial designs. 

## 2. Experimental Section

### 2.1. Construction of VPM1002

VPM1002 was constructed by a single step that disrupted the *ure*C locus by insertion of a *hly* gene coding for an LLO expression cassette. The leader sequence of Ag85B was included in front of LLO to allow for secretion of the protein [4]. For construction of the vector used to generate VPM1002, flanking regions of the *ure*C gene were introduced upstream and downstream of the hygromycin B resistance cassette of the mycobacterial recombination vector. The LLO expression cassette was then inserted between the 3'-region of *ure*C and the antibiotic marker to result in the final construct, which was electroporated into the parental strain to allow for homologous recombination. Individual hygromycin resistant clones were selected from Middlebrook 7H11 agar containing 80 µg/mL hygromycin B. Genetic modifications were confirmed by PCR, Southern blotting and deep sequencing.

### 2.2. VPM1002 Manufacturing

VPM1002 was adapted to animal-free Sauton’s medium to create a Master Seed Bank (MSB) and used to produce the Working Seed Bank (WSB). Bulk Drug Substance (BDS) was produced using three 200 mL cultures grown in shake flasks containing Sauton’s medium. The contents of the flasks were used to inoculate a 30-L fermenter containing Sauton’s medium. After meeting the desired growth OD_600_, contents were harvested, cross filtered, and the concentrated bulk was combined with formulation buffer. The material was then filled into 2 mL amber serum vials and lyophilized, stoppered, crimp-sealed, labeled and stored at 2–8 °C and used for the following nonclinical studies. 

### 2.3. VPM1002 Protection Study in Mice

Female BALB/c mice were vaccinated subcutaneously with 1 × 10^6^ viable bacilli of BCG Danish-SSI 1331, VPM1002 or saline. After 90 days mice were infected with a low dose (200 CFUs) of Mtb H37Rv per animal using a Glas-Col inhalation exposure system. At 30, 60 and 90 days post-challenge, six mice per group were sacrificed, and lungs and spleens homogenized in saline with 0.05% Tween 80 and plated in serial dilutions onto Middlebrook 7H11 agar. Plates were incubated for 3–4 weeks at 37 °C prior to enumerating viable bacterial colony forming units (CFU). Experiments had ethical clearance by the Landesamt fuer Gesundheit und Soziales Berlin under permit number G0307/11.

### 2.4. VPM1002 Guinea Pig Toxicology Study

The purpose of these studies was to obtain information on the toxicity of VPM1002 in male and female guinea pigs after single subcutaneous application of the test item followed by periods of 6 to 26 weeks and dosages up to 50-fold human target dose (HTD). The HTD was considered 1–4 × 10^5^ CFU and a total of 6 female and 6 male guinea pigs per experiment were randomly allocated to two treatment groups of 6 animals each (3 female and 3 male animals per group). Body weight was determined three times a week during the observation period, animals were checked three times a week for clinical findings and mortality was recorded daily. At the end of the observation period all animals were necropsied and macroscopically altered organs were evaluated histopathologically.

### 2.5. VPM1002 Newborn Rabbit Safety Study

Information derived from this study serves to indicate test-item-related local intolerance reactions, signs of systemic toxicity, mortality and mycobacterial load in target organs. Newborn rabbits were vaccinated subcutaneously within the first 2 days after colostrum feed with either one human target dose (1–4 × 10^5^) of VPM1002, BCG Danish-SSI 1331 or saline followed by an observation period of 90 days, with samples collected 10, 21 and 90 days post-vaccination for gross necropsy and histopathology. Special attention was paid to the local tolerance at the injection site. Target organs were investigated for their mycobacterial load by evaluating the number of CFUs in tissue samples. VPM1002 colonization in target organs was stained and assessed by microscopy. The body weight of each newborn rabbit was monitored on the day of administration, twice weekly until the end of week 2, and weekly from week 3 onwards

### 2.6. Construction of AERAS-422

The plasmid vector, pRC131, containing TB antigens Ag85A, Ag85B and Rv3407, was created using the *ori*M and *panCD* genes from plasmid pRC128 and the antigen cassette from plasmid pRC102. pRC128 was first digested with *Bam*HI & partially digested with *Xba*I. The 7.8 kb fragment (plasmid backbone) was ligated with the 2.7 kb antigen cassette generated by PCR from pRC102. pRC131 was digested with *Pac*I to remove the antibiotic marker and self-ligated. The self-ligated, purified plasmid was electroporated into AERAS-413 (a pantothenate auxotroph of AERAS-401 [8]) and selected on antibiotic-free, 7H10 agar plates without pantothenate supplementation. 

### 2.7. AERAS-422 Manufacturing

AERAS-422 was adapted to animal-free medium to create an Accession Cell Bank (ACB) and used to produce the Master Cell Bank (MCB). The Bulk Drug Substance (BDS) was produced using three MCB vials grown in shake flasks containing 7H9 medium. The contents of the flasks were used to inoculate a 20-L fermenter containing 7H9 medium. After meeting the desired growth of OD_600_ 5 ± 1, contents were harvested, centrifuged, and the cell pellet was resuspended and stored in vapor phase liquid nitrogen. To produce a final fill lot, vials of concentrated BDS were thawed, combined with formulation buffer, and dispensed into 2-mL amber serum vials. The contents of the vials were lyophilized, stoppered, crimp sealed, labeled and stored at ≤−65 °C and used for the following nonclinical studies. 

### 2.8. Immunogenicity of AERAS-422 in Non-Human Primates (NHPs)

Female Chinese rhesus macaques, between 2–5 years of age, were used in this study. Animals were housed at Advanced Biosciences Laboratory, Inc (Rockville, MD, USA), in accordance with the standards of the American Association for Accreditation of Laboratory Animal Care. Animals were randomized into groups including saline (n = 4), BCG Danish-SSI 1331 (n = 6) and AERAS-422 (n = 6) and were immunized intradermally with 100 µL of saline, BCG Vaccine SSI (1 × 106 CFUs) or AERAS-422 (1 × 106 CFUs). Animals were bled at weeks 0, 6, 8 and 12 and PBMCs were isolated by standard Ficoll-Hypaque method [10]. Intracellular cytokine stimulation and staining was then performed as previously described [11] with LIVE/DEAD viability dye (Violet, Invitrogen) and the following antibodies: CD3-APC-Cy7, CD4-PerCP-Cy5.5, CD8-APC, IFNγ-FITC, IL2-PE, TNFα-PE-Cy7, CD14-Pacific Blue, CD16-Pacific Blue (all from BD). Samples were acquired in a BD LSRII flow cytometer and analyzed using FlowJo Software. Cells were gated on FSC-A *vs*. FSC-H to exclude doublets. CD3 T cells were then gated against V450 (a dump gate including viability dye, CD14 and CD16), followed by gating on CD4 and CD8 T cells. 

### 2.9. Mouse Protection Study of AERAS-422

In a mouse protection study approved by the institutional animal care and use committee of the Center for Biologics Evaluation and Research, C57BL/6 mice were vaccinated subcutaneously with saline, 5 × 10^6^ CFUs of BCG Danish-SSI 1331 or AERAS-422 as described previously [12]. After 8 weeks, the vaccinated mice were challenged by aerosol with 100–200 CFUs of the HN878 strain of Mtb using a Glas-Col inhalation chamber. Mice were sacrificed at 1, 3 and 5 months post-challenge. Lungs and spleens of individual mice were homogenized separately in 5 mL normal saline plus 0.05% Tween-80 using a Seward Stomacher 80 blender (Tekmar). The homogenates were diluted serially and plated on Middlebrook 7H10 agar containing thiophene-2-carboxylic acid (2 µg/mL) to prevent growth of BCG. Lung tissues were processed for histopathology using standard paraffin fixation, sectioning, and H&E staining. The H&E stained lung sections were evaluated using computer-based histopathology analysis as described previously [13].

### 2.10. AERAS-422 Safety Studies

Six- to 8-week-old female SCID mice (Charles River, Wilmington, MA, USA) were immunized with 100 µL (5 × 10^6^) of BCG Danish-SSI 1331(Staten Serum Institute, Denmark; diluted in Sauton’s medium), AERAS-422 and AERAS-401 (Aeras, Rockville, MD, USA; diluted in saline via tail vein injection). Mice were sacrificed when moribund; defined by severe lethargy, hunched with ruffled fur, loose skin or other signs of severe distress in line with FELASA recommendation on limiting clinical signs.

For repeat dose toxicity study, guinea pigs were injected intradermally with approximately 10 times greater than the current recommended BCG dose given to humans (~2–8 × 10^5^ CFUs/0.1 mL adult dose). To test excessive dermal reactivity in line with European pharmacopeia and WHO recommendations [14,15] for assessing BCG vaccines, guinea pigs were injected intradermally with BCG Danish-SSI 1331 (8 × 10^4^) or AERAS-422 (5 × 10^6^) and observed for adverse effects. Testing for freedom from virulent mycobacteria was performed to detect the presence of virulent mycobacteria in the lyophilized vials of AERAS-422 to be used in human clinical trials. Guinea pigs were injected subcutaneously with two doses of AERAS-422, low (2.5 × 10^7^) and high (1 × 10^8^), to achieve a target dose of 10 and 50 times the highest anticipated clinical dose. General safety of AERAS-422 was evaluated in both mice and guinea pigs with a five times higher dose (mice; 5 × 10^6^) and a 50 times higher dose (guinea pigs; 2.5 × 10^6^) than the highest anticipated human dose.

## 3. Results and Discussion

### 3.1. Novel Recombinant BCG—VPM1002

The rBCG currently in the most advanced clinical studies is VPM1002. VPM licensed the vaccine candidate from the Max Planck Society and has sponsored the preclinical-clinical development to the current clinical phase IIa studies in neonates. This novel construct was produced by integrating the *hly* gene from *L. monocytogenes* coding for LLO expression into the genome of BCG [4]. LLO is a non-enzymatic toxin with pore forming functionality. In contrast to other pore-forming bacterial toxins, LLO provides two safety features intended to prevent damage or death of host cells: (1) The pore forming functions of the protein is restricted to acidic pH with optimal activity at pH 5.5 [16]; (2) LLO also carries the PEST amino acid sequence that directs the protein to phagosomal degradation upon appearance in the host cell’s cytosol [17,18,19]. In contrast to BCG, which is restricted to the phagosome and thereby considered to be limited to MHC II antigen presentation, the LLO activity of VPM1002 allows bacterial antigens to enter the cytosol and gain access to the MHC I pathway [4,5]. MHC I antigen presentation and subsequent stimulation of CD8 T cells is considered to more closely resemble the natural mechanism of protection against TB which, through the *esx1* region, allows for phagosomal escape [20], and is thus believed to be the appropriate means for improving the induction of immunity by this modified BCG [21]. The targeted integration of this gene into *ure*C also serves to inactivate BCG’s gene for urease (*ure*C). Urease catalyzes the hydrolysis of urea into carbon dioxide and ammonia, thus creating a basic environment. Inactivation of this gene is necessary since listeriolysin is optimally active under acidic conditions, as previously mentioned. Both modifications were engineered in one approach by the insertion of *hly* into the *ure*C locus, thus disrupting the urease sequence and deleting its activity [5]. Studies have demonstrated that these modifications lead to enhanced apoptosis and enhanced MHC class I antigen presentation [5]. The final VPM1002 construct is sensitive to antibiotics commonly used in the treatment of mycobacterial infection, including isoniazid, rifampicin and ethambutol. The correct genetic nomenclature of VPM1002 is recombinant *M. bovis* BCG *∆ureC::hly* (genetic background Danish, subtype Prague).

**Figure 1:**
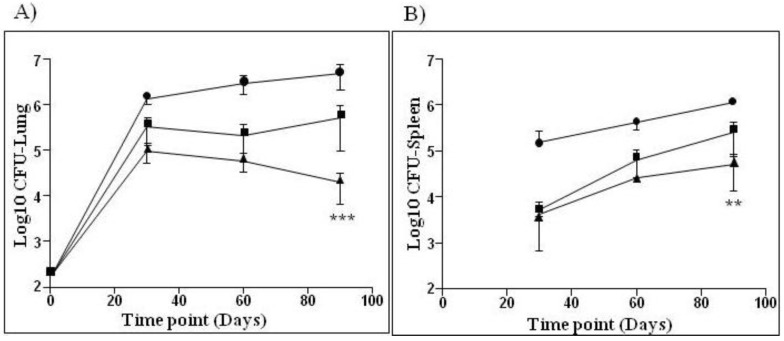
Efficacy study in mice comparing VPM1002 with BCG Danish-SSI 1331. Balb/c mice were immunized subcutaneously with 10^6^ BCG SSI (■), VPM1002 (▲) or with PBS (●). After 90 days, animals were infected via aerosol with 200 CFUs of Mtb (H37Rv) per mouse. Bacterial burden of lung (**A**) and spleen (**B**) was determined at 30, 60 and 90 days post-challenge. Data is shown as mean and standard deviation (n = 6 per group) of one representative experiment out of three. One-way ANOVA and Tukey’s multiple comparison was used to determine statistical significance of VPM1002 as compared to BCG SSI (*p* < 0.05).

VPM1002’s preclinical proof of concept for efficacy and safety has been analyzed in 10 studies comprising approximately 600 animals. In general, all studies were performed using BCG Danish, sub type Prague vaccine as a baseline for vaccine induced responses. For efficacy, mice were vaccinated with VPM1002 or BCG and subsequently challenged by aerosol, using either the laboratory strain Mtb H37Rv or a clinical isolate of Mtb. Efficacy was determined by enumerating viable Mtb colony forming units (CFUs) in the lung and spleen. Preclinical testing of VPM1002 in mice demonstrated better protection from Mtb infection compared to BCG SSI (Figure 1). The immune response induced was sustained with bacterial burdens in lungs and spleens of VPM1002-vaccinated mice consistently below the BCG Danish-SSI 1331 control and the difference became statistically significant at 90 days post-infection. In all 10 studies, VPM1002 demonstrated better protection against the TB challenge when compared to the BCG positive control.

The safety of VPM1002 was evaluated in a number of animal models and in three different species. Interferon-gamma (IFNγ) knock-out mice served as a model for evaluating the risk of disseminated infection with BCG and VPM1002 vaccines in severely immunocompromised humans, since BCG infection has been related to deficiencies in the IFNγ signaling pathway. No VPM1002-vaccinated animal died during the observation period of 105 days in contrast to BCG (one animal died), and no adverse effects on animal health were observed. However, both VPM1002 and BCG induced local reactions at the site of vaccination.

The target product profile for clinical development of VPM1002 considered HIV-infected individuals, therefore further safety studies were performed in SCID mice which lack an adaptive immune system. As described in Grode *et al*., 2005 [5], following administration of a 10-fold human target dose (*i.e*., 5 × 10^6^ CFU per animal), the severity and incidence of findings were similar or less severe than the single human dose of the reference BCG strain (given at 5 × 10^5^ CFU). Bridging studies with cGMP-manufactured vaccine showed comparable levels of safety in SCID mice.

Three single-dose toxicity studies were performed in guinea pigs with follow-up periods of 6 to 26 weeks and dosages up to 50-fold human target dose (HTD). In general, weight gain (a highly sensitive marker for TB in guinea pigs) was similar in all treatment groups and in the range of normal variation known for this species. No guinea pig died prior to study end and no lesions typical for TB or related mycobacterial infections were observed during necropsy or histopathological evaluation. Small white spots (approx. 1 mm) were seen on the livers of some animals from all groups (VPM1002, BCG and saline). Histopathology of those spots revealed cell necrosis and hydropic degeneration in both BCG and VPM1002. The pathology seen by microscopy and the naked eye following vaccination with VPM1002 was less than that seen with BCG and no viable bacilli were detected in lung, liver or lymph nodes as observed by culture, although PCR revealed a similar systemic spread of both VPM1002 and BCG nucleic acid in the above mentioned organs. 

**Figure 2:**
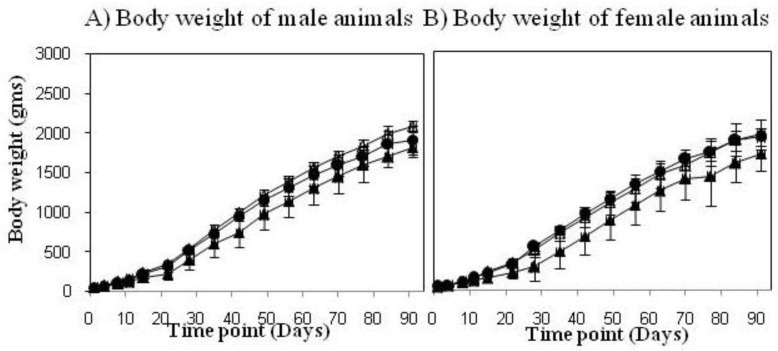
VPM1002 vaccine safety study in newborn rabbits. Weight development of male (**A**) and female (**B**) new born rabbits was evaluated up to 90 days post-vaccination in three treatment groups (Group 1: control 0.9% saline (△), group 2: 1–4 × 10^5^ CFUs BCG (▲) and group 3: 1–4 × 10^5^ CFUs VPM1002 (●) per animal).

In a safety model using new born rabbits and comparing VPM1002 to BCG (both human target/standard dose), no mortality was observed. Macroscopically and histologically, all animals showed clear infectious reactions, which are expected at the 2-week time point post-immunization with a live BCG vaccine. A preclinical study in newborn rabbits was also performed to compare VPM1002 with BCG for safety, including bio distribution of the bacteria 90 days post-vaccination. The body weight of male and female animals vaccinated with BCG (control) did not gain the same amount of weight compared to animals treated with 0.9% saline. No influence on body weight was noted for the animals treated with VPM1002 (Figure 2). Also there were no local adverse reactions or changes in behavior, external appearance was noted. In addition, all critical assays testing the virulence of VPM1002 met all the requirements of the European Pharmacopeia recommendations for BCG [14]. 

### 3.2. Novel Recombinant BCG—AERAS-422

**Figure 3:**
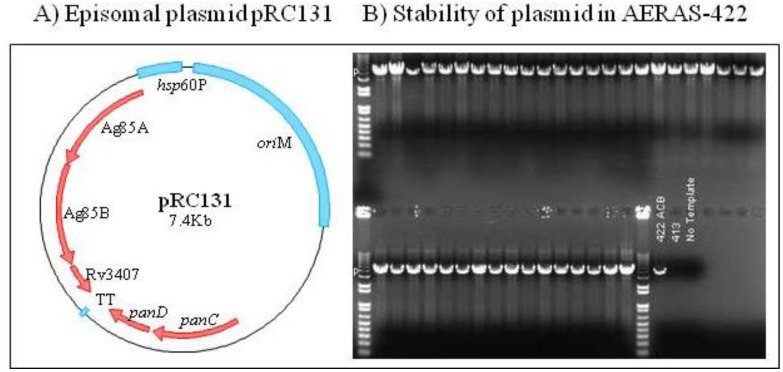
Genetic analysis and stability of AERAS-422 plasmid. (**A**) Schematic representation of the complementation plasmid in AERAS-422 expressing Ag85A, Ag85B and Rv3407c, as well as the complementing *pan*CD gene under its own promoter. (**B**) The stability of the plasmid was tested by plating a sample of AERAS-422 on 7H10 plates without pantothenate supplement and screening 40 colonies using PCR for the presence of the antigen cassette.

A second example of a novel rBCG vaccine that has progressed into clinical studies is the AERAS-422 vaccine. The parent strain (BCG Danish-SSI 1331) modified to express PFO, AERAS-401, has been described previously [8]. Using specialized transduction, the *pan*CD genes coding for pantothenate synthase were deleted [22], rendering AERAS-401 auxotrophic for pantothenic acid (vitamin B-5). This allowed for *pan*CD complementation, which helped to retain the episomal plasmid containing two classical Mtb antigens (Ag85A and Ag85B) and one latency-associated antigen (Rv3407), without the need for an antibiotic marker. The overexpression of these antigens by this new rBCG strain, AERAS-422, was confirmed by Western blot analysis using specific antibodies to the Mtb antigens. Antigen expression was also stable after infecting cells from the human macrophage-like cell line, THP1, and plating at different time points and checking colonies for the presence of genes coding for the above mentioned antigens using PCR (Figure 3). 

**Figure 4:**
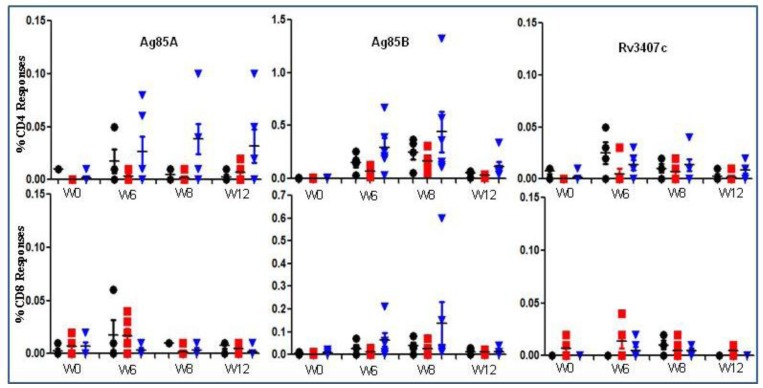
Immune responses to AERAS-422 following immunization of non-human primates. PBMCs from Rhesus macaques vaccinated with either (5 × 10^6^) BCG Danish-SSI 1331 or AERAS-422 were isolated and stimulated with Ag85A, Ag85B or Rv3407c peptides. Intracellular cytokines were examined at weeks 0, 6, 8 and 12. The data is the percent of total CD4^+^ or CD8^+^ T cell responses making IFN-γ, TNF-α, or IL-2 alone or in combination following background (DMSO stimulation) subtraction. Groups shown are: ●—Saline 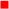
—BCG SSI and 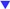
—AERAS-422. Bars show the Mean ± SD.

Both NHPs [23,24] and mice [25] provide a useful model for immunological testing of vaccines due to their homology with humans, and the availability of a broad range of immunological reagents for the examination of immune responses. AERAS-422 was tested for immunogenicity in NHPs and protective efficacy in the mouse model of TB. As shown in Figure 4, AERAS-422 induced considerably higher CD4 responses against each of the antigens and greater CD8 responses against Ag85B only when compared to parental BCG in NHPs. While these increases did not reach statistical significance, the data present a clear trend toward increased immunogenicity. Responses against Rv3407 remained below the limit of detection for this assay.

**Figure 5:**
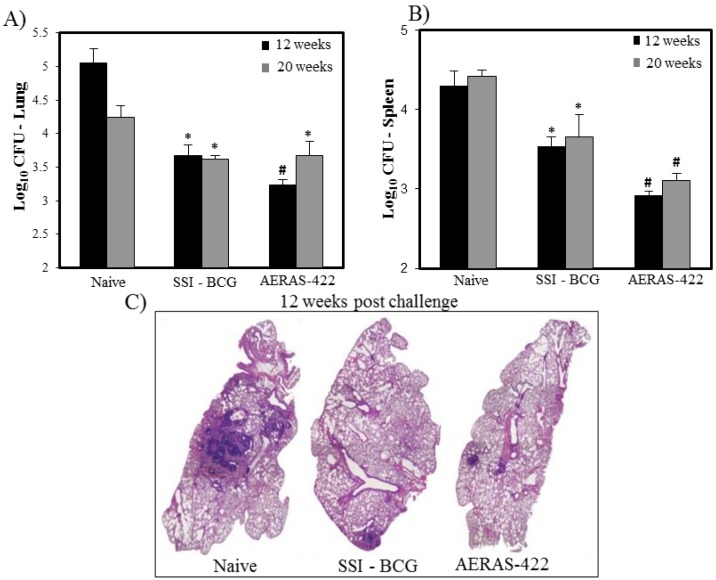
Bacterial load and lung histopathology following immunization with AERAS-422 and challenge of C57BL6 mice with Mtb HN878. Bacterial load in lungs (**A**) and spleen (**B**) were determined 12 (■) weeks and 20 (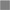) weeks post-challenge. Statistical analysis was performed using the unpaired t test and the symbols represent the following: * = significantly better than naive at *p* < 0.05 and # = significantly better than both naive and BCG at *p* < 0.05. (**C**) Histopathology at 12 weeks post-challenge demonstrating fewer granuloma-like lesions and more open alveolar space in lungs of mice vaccinated with both BCG Danish-SSI 1331 and AERAS-422.

The protective efficacy of AERAS-422 was evaluated in a mouse challenge model. AERAS-422 showed significantly better protection than animals injected with saline (naïve) or BCG in lungs 12 weeks (*p* < 0.05) post-challenge and in spleens at both 12 and 20 weeks (*p* < 0.05) post-Mtb challenge (Figure 5A,B). Using computer-based histopathology analyses, lung sections showed significantly less granuloma-like lesions in both BCG Danish-SSI 1331 (17.6 ± 1.4) and AERAS-422 (20.3 ± 2.5) when compared to naïve mice (48.0 ± 2.5) at 20 weeks post-challenge (Figure 5C).

**Figure 6:**
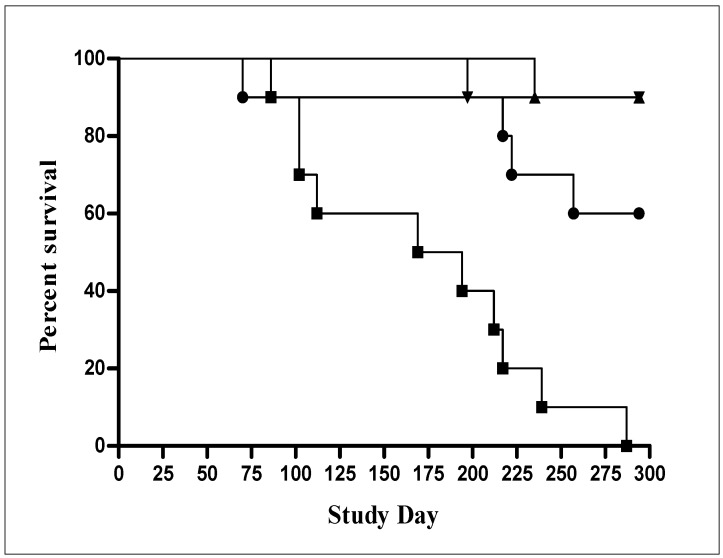
Survival study in SCID mice comparing AERAS-422 strain with BCG parent strains. SCID mice (10 mice/group) were immunized subcutaneously with high doses (5 × 10^6^) of BCG Danish-SSI 1331 (■), AERAS-401 (●) and AERAS-422 (▼) and compared with a naïve group (▲).

Preclinical safety of AERAS-422 was investigated using a SCID mouse model of infection. In this study, 100% (10/10) of mice immunized subcutaneously with BCG SSI died within the 300 day observation period, while 9 out of 10 mice immunized with AERAS-422 survived as did the naïve control group (Figure 6). As mentioned above, AERAS 422 is derived from AERAS-401 [8] a strain which contains PFO but not the Mtb antigen cassette and AERAS-422 immunized mice also survived significantly better than mice immunized with this parent strain where 5 of 10 mice died. Log-rank analysis of the survival curves comparing BCG Danish-SSI and AERAS-422 yielded a *p* value = 0.0002. It may be that the high level of antigen overexpression from the plasmid, in addition to expression of the foreign PFO protein, alters the response to the rBCG such that it was contained by the innate immune system present in SCID mice. 

AERAS-422 was produced as cGMP compliant material using qualified manufacturing procedures. For toxicological evaluation and release of the cGMP material, acute toxicity, repeat dose toxicity, skin test reactivity (PPD) and freedom from virulent mycobacteria studies were performed. Guinea pigs were used for all of these studies due to their sensitivity to TB [26,27]. In repeat dose toxicity no adverse effects were observed and all animals survived to the scheduled termination (day 7 post-immunization) with no changes in appearance, behavior or body weight. The potency release assay, which was done by tuberculin skin testing with PPD after immunization, demonstrated the biological activity of the vaccines. Finally, freedom from virulent mycobacteria also showed no evidence of tuberculosis following immunization.

### 3.3. New Standards for Assessing the Quality, Safety and Efficacy of Recombinant BCG Vaccines

The World Health Organization (WHO) Expert Committee on Biological Standardization has recently revised the “Recommendations to Assure the Quality, Safety and Efficacy of BCG Vaccines”, which is used by global manufacturers of BCG vaccines [15]. Although important for evaluating current BCG vaccines, new vaccine strains have been constructed using novel molecular techniques and have, in some cases, been manufactured using methods that differ from the traditional methods, for example VPM1002 and AERAS-422 vaccine candidates. New recommendations will therefore be required for general manufacturing and vaccine testing methods, as well as for preclinical and clinical testing. Unlike current BCG vaccines, certain new rBCG vaccines are being produced in facilities not dedicated to BCG production alone. Recombinant BCG vaccines generated by novel molecular techniques require tests for attenuation and possible reversion, persistence, plasmid retention and genetic stability, along with antibiotic sensitivity to frontline treatments. Preclinical safety tests use models of immunosuppression such as SCID and knock-out mouse models to predict the safety of these vaccine candidates intended for use in humans with HIV and other immunocompromised conditions. Clinical trials will need to address issues of safety and efficacy in healthy volunteers and in patients infected with Mtb and HIV. In addition, studies will be carried out using live vaccines as a “prime” and potentially use subunit vaccines as a “boost” to the prime. Although not a regulatory requirement, before proceeding past phase I human safety studies it may prove useful to perform efficacy studies in non-human primate TB challenge studies to investigate prime-boost regimens and, if possible, to compare different recombinant BCG vaccines. For all these reasons, additional WHO recommendations could provide an authoritative framework indicating new standards for assessing the quality, safety and efficacy of new live vaccines for TB. Table 1 lists new tests that may need to be implemented for new live TB vaccines compared to the current tests used for traditional BCG vaccines. Many of these tests may also be used for live attenuated Mtb vaccines such as MTBVAC [28] which has recently entered human phase I studies although additional safety testing such as in non-human primates may need to be performed.

**Table 1:** Testing strategies for new live TB vaccines compared with traditional tests.

Test	Comments	New TB vaccines *	Traditional BCG vaccine **
**Identity**	For master, working seed and final lots	Multiplex PCR, sequencing	Microbiologic methods
**Safety**	For bulk and/or final lots	Tests for attenuation, persistence, lack of reversion to virulence	General safety test in mice and guinea pigs
**Safety**	Meet GMO Standards	Evidence for lack of shedding of live organisms in animals	NA
**Freedom from virulent Mtb**	For master, working seed, bulk and/or final lots	Multiplex PCR for live vaccine identity and animal safety studies	Guinea pig assay
**Antibiotic sensitivity**	Evidence of susceptibility to first line drugs for TB; removal of antibiotic resistance selection markers	MGIT analyses (liquid culture)	Solid media culture analyses
**Viability**	For bulk and final lots	ATP assay live/dead ratio	Solid media culture
**Potency**	For bulk and final lots	Immune-biological assay (to be defined)	Viability
**Stability**	For bulk and final lots	Immune-biological assay (to be defined)	Viability/Moisture. Thermal & real time stability. Intradermal skin test in guinea pigs
**Toxicology**	For investigational lots	Immune-relevant toxicology test (to be defined)	Necropsy analyses in rabbits
**Lot release tests**	New methods may include fermentation process and synthetic media used to culture mycobacteria	Tests for residual contaminants from fermentation	Evidence for no TSA—containing culture media
**Preclinical studies**	To assess safety, immunogenicity & efficacy in animal models	Survival in animal models of immunosuppression. Protection in guinea pigs and/or mice. Safety studies in NHP, (for live attenuated Mtb vaccines). Identification of immunological markers	NA
**Validation of facilities**	Manufacturing facilities for live TB vaccines	PCR of individual products and cleaning, validation when campaigning products other than TB vaccines; Certain live attenuated vaccines may require dedicated facilities	Dedicated facilities, equipment and staff

* Many of these assays are still under development and have not been standardized; ** Traditional tests as described here may be used in the characterization of new TB vaccines.

## 4. Concluding Remarks

### 4.1. Challenges for Recombinant BCG Vaccines

As previously described [29], investigators face a number of challenges in the rational development of new BCG vaccines to demonstrate that they are safer and more efficacious than the current BCG vaccine. New rBCG approaches should include a novel formulation of BCG which impacts its potential to elicit more protective immunity directed against antigens found in Mtb while not compromising safety. In the two examples presented here, this includes the addition of a heterologous bacterial lytic gene which may promote antigen presentation to the host immune system [5,30] and the expression of Mtb antigens known to elicit protective immune responses in animal models [8]. As rBCG vaccines will be considered a genetically modified organism (GMO), studies to characterize them should meet regulatory requirements relevant to live vaccines such as attenuation, non-reversion, persistence, and shedding. The greatest challenge in rBCG development is demonstrating superior immunogenicity and protection that may be relevant to humans using preclinical animal models. Since there is no known “correlate of protection”, investigators are left measuring immune responses that are only thought to be related to TB protection [31]. In animal protection *vs*. challenge experiments, which are commonly the most important factor in deciding whether to move a vaccine candidate forward, investigators have used various BCGs as comparators and various immunization and Mtb strains and challenge strategies [32]. As shown in this article and elsewhere [6], rBCGs can be demonstrated to be superior to BCG comparators in animal experiments but results can differ depending upon experimental variables. It is clear from the experience of a number of investigations [33] that time and effort invested in the development of a novel rBCG should be balanced against potential benefits.

### 4.2. Potential Benefits of New BCG Vaccines

A new rBCG vaccine may be more acceptable (marketable) to the global community, as long as it can be shown to be safer and more efficacious than BCG, and if the cost is comparable or lower than the currently available BCG. Unlike traditional BCG vaccines, novel vaccines offer several advantages including:

- new well-characterized products manufactured by state-of-the-art technologies
- shown to be safe and effective in contemporary clinical trials compared to currently used traditional BCG vaccines
- may serve as excellent prime vaccines for novel booster TB vaccines currently under development.

A robust process for developing candidate products that have undergone extensive preclinical studies such as those described in this article would facilitate acceptance with regulatory agencies for clinical development. In general, it would provide a better-defined product than BCG licensed decades ago by traditional methods. Table 1 outlines testing strategies that could differ between new live BCG vaccines and traditional BCG vaccines. The possibility for an updated regulatory review and product approval of new BCG based vaccines offers an opportunity to develop more rigorous characterization of BCG vaccines and to apply new tests for characterizing purity, safety and, particularly, potency. In historical studies, BCG vaccines have shown variable efficacy in different populations and age groups and the accepted efficacy of currently used BCG vaccines is estimated from meta-analyses of previous clinical trials and case control studies [34,35]. PPD/TST conversion correlates with BCG vaccination with respect to vaccine uptake, but does not appear to correlate well with clinical efficacy [36], although this test has been used for many years by regulatory agencies for the licensure and quality control of BCG. Currently, the identification of relevant biomarkers for immunogenicity and protection by TB vaccines is a high priority [37,38] and it is hoped that some biomarkers may become available for testing in rBCG clinical phase II studies, and may also be tested as correlates of vaccine efficacy or protection in clinical phase III studies. These biomarkers may therefore provide a pathway for regulatory agencies to consider alternative data as supportive evidence for more efficient clinical development towards licensure of rBCG candidate vaccines.

Current BCG vaccines can be relatively reactive vaccines that cause ulceration, scarring and lymphadenopathy, as well as serious adverse effects from dissemination in immunocompromised infants [39,40,41]. Robust preclinical safety and human safety studies in the target populations will be critical for licensing a new BCG vaccine. Current WHO/EPI policy calls for immunization of infants at birth who are not HIV-infected, and there is concern related to immunizing infants with mothers who are HIV+ [2]. Safety studies are a major focus of the preclinical studies described here, and together with robust clinical data, rBCGs may be shown to be safer to use in a HIV+ high risk population. A thorough set of safety data demonstrating a lower incidence of fevers, anemia or lymphadenopathy could establish the basis for licensure of an rBCG vaccine if accompanied by robust post-licensure surveillance. Also, since rBCG is similar to BCGs that have a long history of acceptable safety and use in humans, it may be possible to consider a licensure strategy with smaller or fewer efficacy studies, particularly if preclinical immunological data and clinical safety study data is robust and the sponsor commits to large clinical phase IV post-marketing studies. 

A strategy presently employed by sponsors of TB vaccine candidates in clinical trials is the use of TB subunit vaccines to boost subjects previously vaccinated with BCG. However, several of these antigens are expressed by BCG at low levels or not at all [38]. Development of an rBCG expressing specific Mtb antigens may provide the opportunity to match a prime immunization with the same antigens expressed in the subunit boost vaccine [32]. This offers the opportunity to generate rBCGs overexpressing Mtb antigens that are found in potential boosting vaccines such as MVA85A which is currently being evaluated in a phase IIB efficacy study in BCG vaccinated infants [42]. An NHP study using AERAS-422 boosted by a viral-vectored TB vaccine [43] is an example of an immunogenicity proof of concept preclinical study performed to address the potential of the matched BCG prime-boost concept. However, the ability of an rBCG prime-boost strategy to enhance efficacy remains unclear since some TB vaccine studies have shown little or no booster effects following a BCG prime [44].

In this report, we have provided examples of testing strategies that have been successfully applied to two new rBCG vaccines that have entered human clinical trials [45]. Although not without risks, there are a number of potential benefits that may result from introducing new rBCG vaccines. These include a live replacement TB vaccine that is safer to use in all populations, including HIV+ populations, and be more effective either alone or as part of a prime-boost strategy in controlling adult pulmonary TB disease. The TB community continues to assess new live mycobacterial constructs that will be evaluated over the next decade with the goal of contributing to a meaningful reduction in the global TB epidemic.
